# Fractures with complex fracture patterns are associated with increased rate of subsequent conversion to total knee arthroplasty after a tibial plateau fracture: an observational cohort study of 12,012 patients from the Swedish Fracture Register

**DOI:** 10.1186/s43019-025-00279-0

**Published:** 2025-06-06

**Authors:** Fredrik Olerud, Anne Garland, Nils P. Hailer, Olof Wolf

**Affiliations:** 1 Department of Surgical Sciences, Section for Orthopaedics, Uppsala University, Uppsala, Sweden; 2 Ortopedkliniken, Visby Lasarett, S:t Göransgatan 5, 621 55 Visby, Sweden

**Keywords:** Knee, Tibial plateau fractures, Total knee arthroplasty, Trauma, Lower extremity, Arthroplasty

## Abstract

**Background:**

Tibial plateau fractures (TPFs) can be associated with development of significant joint degeneration, which can lead to functional impairment and pain severe enough to necessitate conversion to total knee arthroplasty (TKA). The factors influencing the progression to TKA after TPF, including preoperative fracture and patient characteristics, remain unclear. This study aimed to assess the national conversion rate to TKA following TPF depending on fracture type.

**Patients and methods:**

The cohort consisted of all patients aged 18 years and older at time of injury with a TPF registered in the Swedish Fracture Register (SFR) between 2012 and 2023. The SFR holds information on baseline patient characteristics including fracture classification according to the AO/OTA system. Conversion to TKA was identified through linkage with the Swedish Arthroplasty Register (SAR). Kaplan–Meier survival analyses investigated conversion rate. Cox regression was performed to assess association between fracture type and TKA conversion adjusted for age, sex, and injury energy level. The follow-up period ranged from 0 to 12 years, with a mean of 4.2 years.

**Results:**

A total of 12,012 patients with a mean age of 57 years were included; 63% were women. The observed conversion rate after 5 years was 2.8% in all patients and 4.1% in surgically treated patients. The conversion rate at 5 years was highest in the 65–74 years age group with 5.2%. Fractures with comminuted fracture patterns, particularly AO/OTA 41B3, 41C2, and 41C3, were associated with significantly increased risks of conversion, with adjusted hazard ratios (aHRs) of 2.1 (95% CI 1.3–3.3), 2.3 (1.2–4.5), and 3.2 (95% CI 2.0–4.5), respectively. High-energy trauma did not increase the risk of conversion, nor did sex. Increasing age was associated with an increased risk of conversion up to the age of 84, while age over 85 was not.

**Conclusions:**

Fractures with complex fracture patterns, particularly AO/OTA 41B3, 41C2, and 41C3, were associated with an increased TKA conversion rate following TPF. The conversion rate increased with increasing age, but sex and high-energy injury mechanisms did not affect conversion rate. On a national level, 3% of patients were converted to TKA within 5 years of sustaining a TPF, and 4% of patients treated surgically. This may help surgeons when counseling patients with TPFs.

## Introduction

Tibial plateau fractures (TPFs) are complex injuries that impact the knee joint, often resulting in significant functional impairment and post-traumatic osteoarthritis (PTOA) [3, 6, 8, 9, 15, 20, 22]. These fractures generally occur due to high-energy trauma in the younger population, such as road traffic accidents or fall from heights, or low-energy trauma in individuals with osteoporosis [20]. Despite advances in surgical techniques with locking plates placed in soft-tissue-friendlier approaches treating all sides of the proximal tibia followed by a postoperative care focusing on range of motion [10, 24], many patients face long-term functional impairment and pain that may require further interventions.

Total knee arthroplasty (TKA) is the gold standard treatment for end-stage symptomatic knee osteoarthritis, which also includes PTOA. Although numerous studies including a systematic review have investigated the conversion rate after TPF [5, 7, 23, 25], the factors influencing this progression remain somewhat unclear. Several studies have explored radiographic factors, such as tibial alignment, widening of the tibial plateau, level of comminution, and degree of depression related to joint failure in these patients [1, 4].

Some large registry-based studies report conversion rates of 4.3–7.3%, but lack critical variables such as preoperative fracture classification and patient characteristics that may influence the rate and timing of conversion to TKA [5, 23, 25]. We have previously reported a conversion rate at 2 years of 5.2% after surgical treatment of TPFs from a single-center cohort study [18]. Assessment of preoperative fracture classification revealed higher conversion rates for fracture types with comminuted fracture patterns (AO/OTA B3 and C3).

Although the conversion rate to TKA after a TPF has been previously investigated, the factors influencing which fractures or patient characteristics contribute to an increased risk of conversion remain less well understood. In particular, the association between fracture classification and risk of conversion has not been fully elucidated. A pertinent question in contemporary orthopedic research is whether a TPF with severe articular comminution in a patient with osteoporosis can be successfully managed with open reduction internal fixation (ORIF) while achieving sufficient pain relief and joint function to avoid the need for subsequent TKA.

The primary aim of this study was to assess whether the widely used Arbeitsgemeinschaft für Osteosynthesefragen/Orthopaedic Trauma Association (AO/OTA) classification system provides preoperative prognostic information on the risk of conversion to TKA following a TPF. Secondary aims included evaluating the association between other preoperative factors—such as age, sex, injury mechanism, trauma energy (low versus high), and surgeon experience—with the likelihood of conversion. The goal was to determine whether certain patient subgroups are at a higher risk of requiring TKA after a TPF.

## Patients and methods

**Fig. 1:**
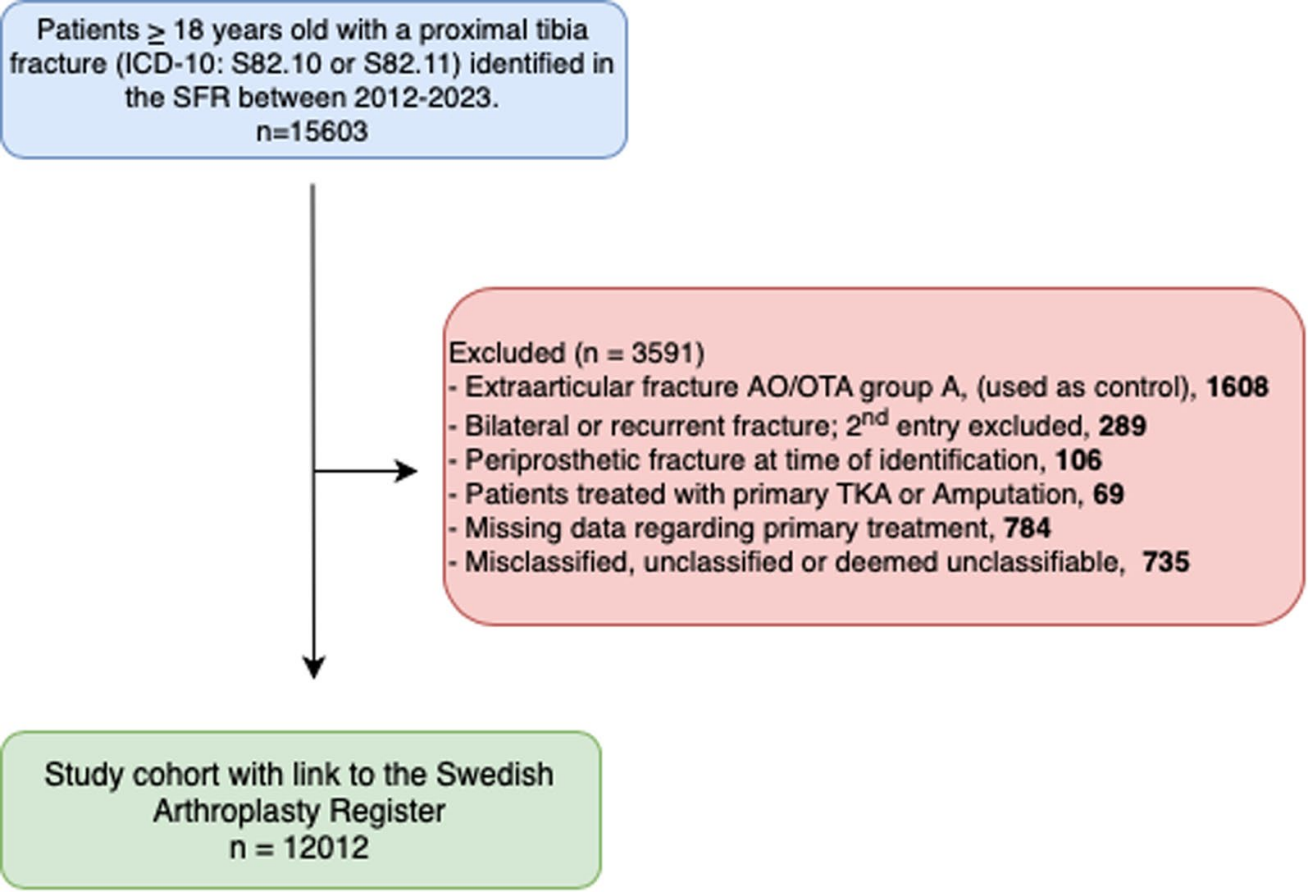
Flowchart of the study population of 12,012 intraarticular tibial plateau fractures after applied exclusion criteria

Using data from the national Swedish Fracture Register (SFR), we identified all patients aged 18 years or older at injury with a registered TPF between 2012 and 2023 (Fig. 1). Patients with extraarticular proximal tibia fractures, AO/OTA group A, were not assessed in this study, but used separately as a control group. In bilateral cases, the second entry was excluded due to challenges with dependency issues in survival analysis [21]. Patients with initial nonsurgical treatment or fracture fixation were included. Patients with other primary treatment, such as amputation, arthrodesis, or primary TKA, were excluded, as they would not be eligible for subsequent conversion. Patients with erroneous registrations and unclassifiable or periprosthetic fractures, as well as patients with missing information regarding treatment, were excluded. The study cohort was subsequently linked on the basis of the personal identity number (PIN) with the Swedish Arthroplasty Register (SAR) to identify conversions to TKA on the injured side until 31 December 2023. The linked register data contained only case numbers, preventing patient identification. Consequently, physician notes and pre- or postoperative radiographs were unavailable for detailed analysis.

This study was conducted in accordance with the Strengthening the Reporting of Observational Studies in Epidemiology (STROBE) guidelines to ensure rigorous methodological and reporting standards.

### Variables in the SFR

From the SFR, we obtained data on patient age, sex, injury mechanism including injury energy type, fracture classification, and primary treatment. Mechanism of injury was recorded using ICD-10 “External Causes of Morbidity” and categorized into four groups: falls, transportation-related injuries, stress fractures, and other causes. Injury energy type was labeled as high energy, low energy, unknown, or not applicable. Data were registered and fractures were classified by the treating physician using the Arbeitsgemeinschaft für Osteosynthesefragen and Orthopaedic Trauma Association (AO/OTA) 2007 classification system in the web-based SFR platform [16, 17]. The tibia fracture classification in the SFR has been validated showing moderate agreement for type specific classification (kappa = 0.56) and substantial agreement for group specific (kappa = 0.75) when compared with an expert group [26]. Experience of main surgeon was classified as orthopedic trauma consultant (> 50% trauma), orthopedic consultant, or resident.

### Outcome measures

The primary outcome of this study was conversion to TKA at 5 years, which was identified through data linkage with the SAR. The SAR provides 98% national coverage and a completeness rate of 96% for primary TKA procedures.

Cumulative incidences of conversion were determined at 1, 2, and 5 years, and at full follow-up. Conversion rates were also compared between fracture types. Secondary outcomes were to assess the association between conversion to TKA and baseline characteristics such as age, sex, injury energy level, and fracture classification. The impact of primary treatment was also assessed, and the association of surgical/non-surgical treatment and conversion to TKA was investigated.

### Statistical analyses

Descriptive statistics were used to describe the study population using mean age (standard deviation, SD) and age was treated as a categorical variable divided into five age groups (< 55, 55–64, 65–74, 75–84, ≥ 85). Cumulative incidence was assessed at 1, 2, and 5 years, and full follow-up periods. Kaplan–Meier survival analysis was performed to assess conversion rate to TKA with patients censored at the end of follow-up or at the time of death, whichever occurred first.

Cox proportional hazards regression was conducted to assess the association between preoperative fracture classification and TKA conversion, both unadjusted (HR) and adjusted (aHR) for age, sex, and injury energy type. 95% confidence intervals (CIs) were used to describe estimation uncertainty. Cox proportional hazards regression was also used to further evaluate the association between TKA conversion and age and injury energy type, respectively.

The influence of surgeon experience on the outcome was assessed using a Cox proportional hazards model adjusted for fracture severity, age and sex, with residents defined as the reference category.

Kaplan–Meier survival analysis and a Cox proportional hazards model were used to assess conversion rate and association between surgical treatment and the risk of TKA conversion.

Type A fractures (extraarticular proximal tibia) were used as a control group in Cox proportional hazards regression models and Kaplan–Meier survival analysis to assess the risk of different fractures requiring TKA conversion. Schoenfeld residuals were calculated and plotted to test proportionality assumptions and ensure model fit.

A two-tailed alpha of 0.05 was used for all statistical tests. Analyses were performed using R software (version 4.3.1) [19].

### Ethics, funding, data sharing, and potential conflicts of interest

Ethical approval for the study was obtained from the Swedish Ethical Review Authority (Dnr 2023–06309-01). The study was conducted in accordance with the ethical principles of the Declaration of Helsinki. No specific funding was received for this study. Data from this study are not freely available. However, data can be extracted from the national registries with an approved ethical application due to the sensitive nature of data from national quality registers. The authors declare no competing interests related to this study.

## Results

### Characteristics of the study population

**Fig. 2:**
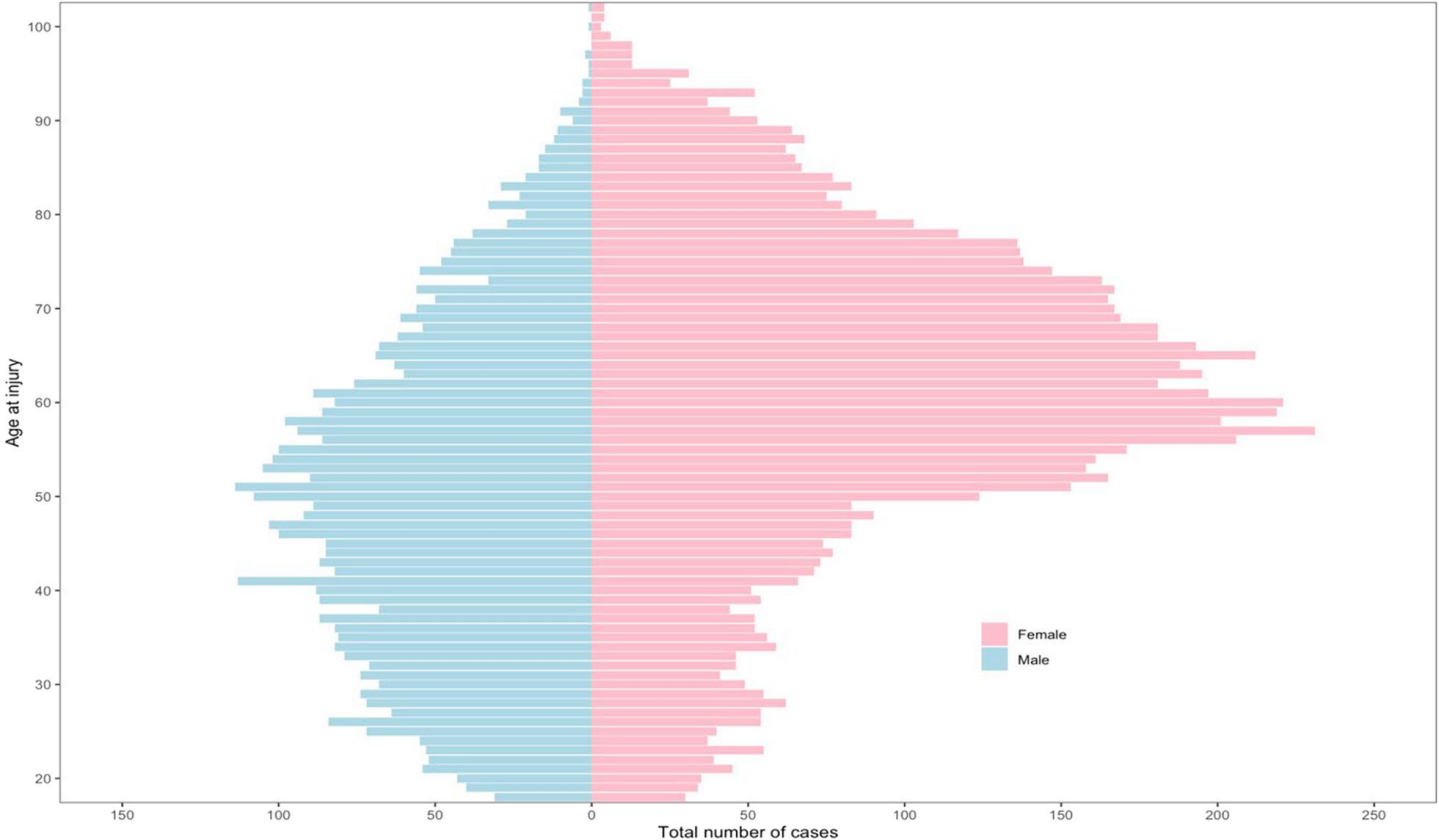
Age and sex distribution of 12,012 patients with tibial plateau fracture

A total of 12,012 patients with a TPF registered in the Swedish Fracture Register (SFR) between 2012 and 2023 were included; 63% were women. The mean age of all patients was 56.6 years (SD 18) (Fig. 2).

**Fig. 3:**
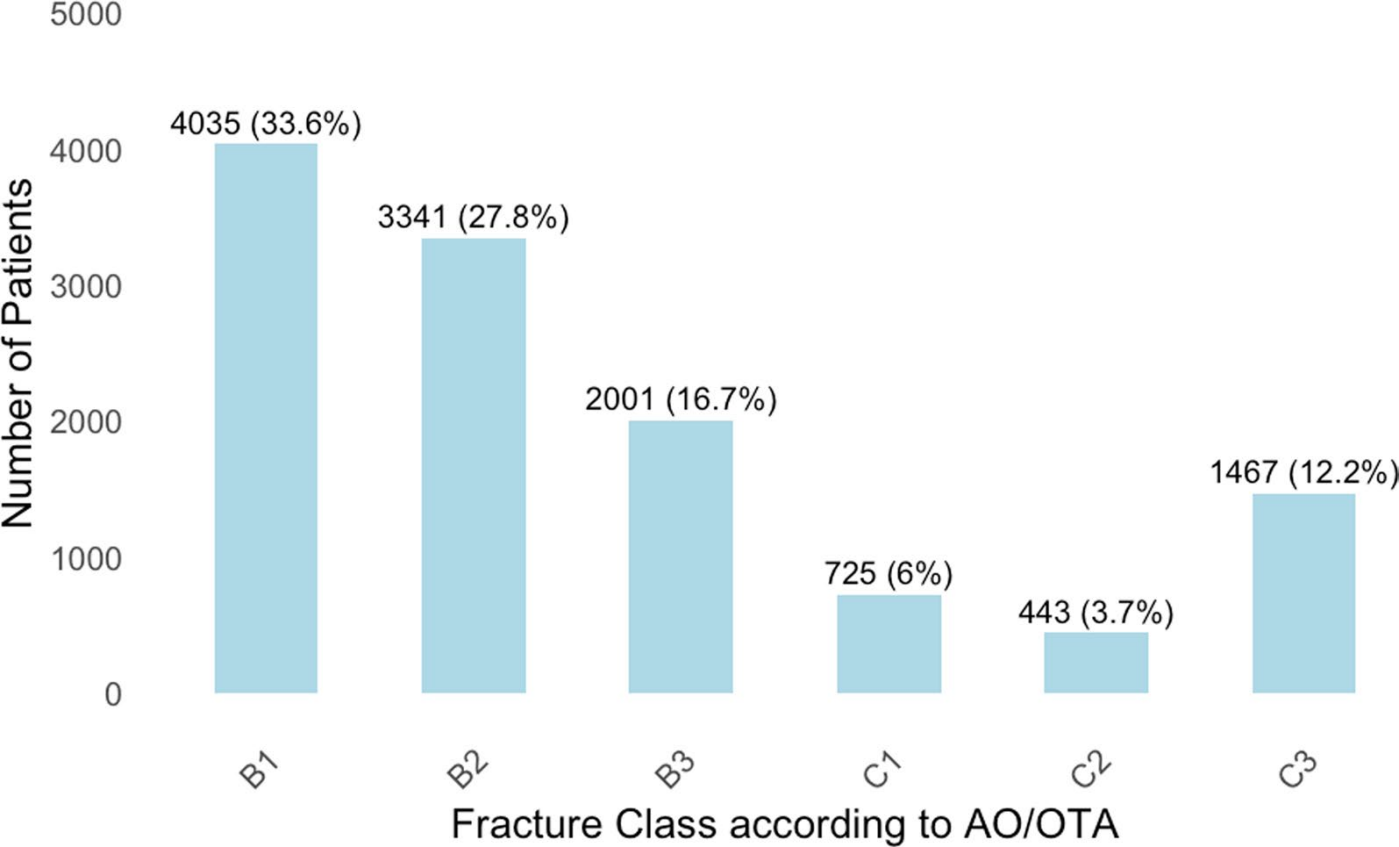
Fracture distribution according to AO/OTA classification in study cohort of 12,012 patients [number of patients (% of cohort)]

**Fig. 4:**
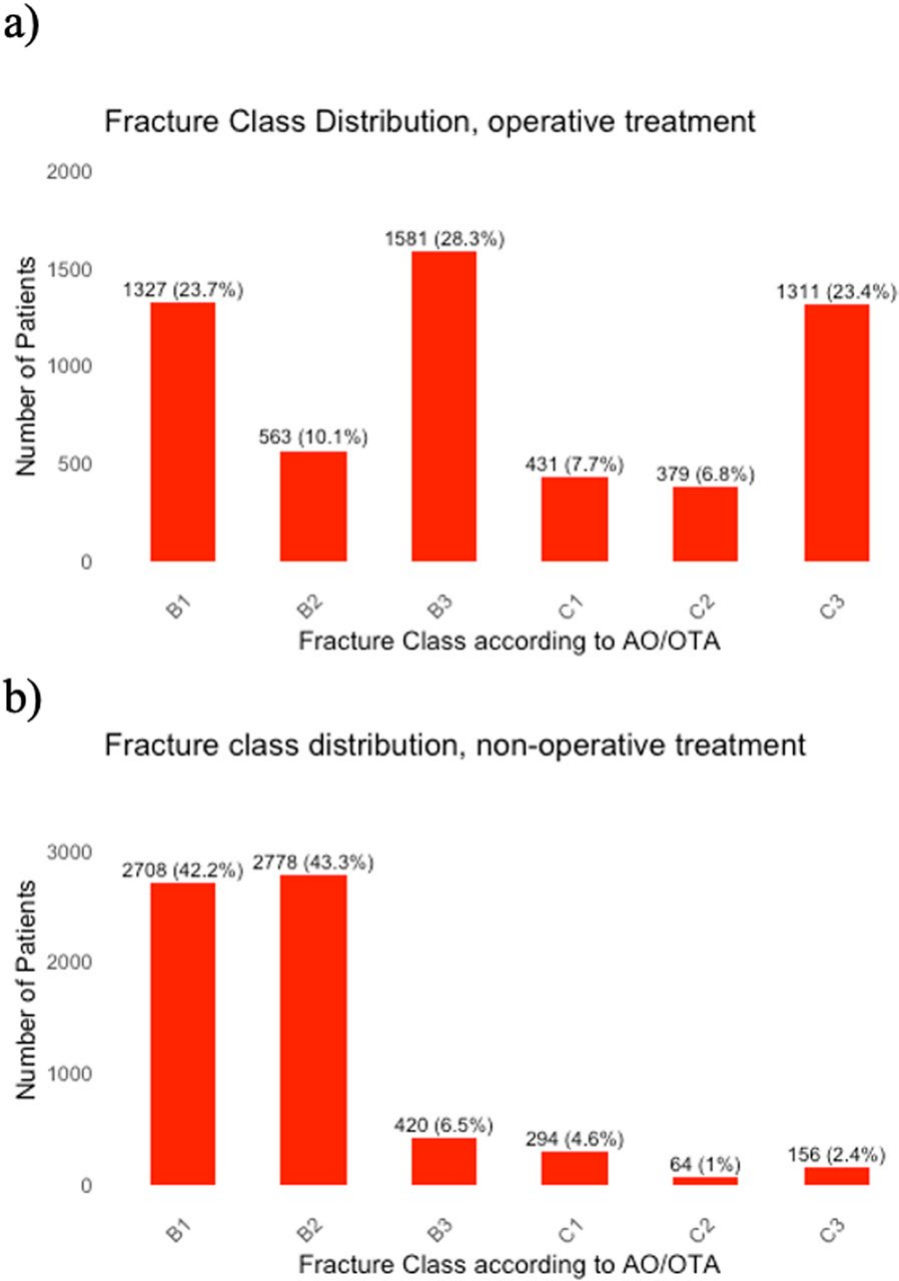
Fracture distribution according to AO/OTA of 12,012 patients with tibial plateau fracture treated. **a** Operatively (*n* = 5592), and **b** nonoperatively (*n* = 6420) as first treatment

The distribution of fractures according to the AO/OTA classification revealed that 34% were classified as type B1, 28% as type B2, 17% as type B3, 6% as type C1, 4% as type C2, and 12% as type C3 (Fig. 3). More than 80% of fractures were B1 and B2 fractures in the nonsurgically treated group compared with a third of fractures in the surgically treated group (Fig. 4). Falls accounted for 62% of the cases, while 23% were related to transportation incidents, < 1% were attributed to stress fractures, and 14% were classified under other causes.

The majority (73%) of fractures were caused by low-energy injuries, and 18% were registered as high-energy injuries; 53% of the TPF were treated nonsurgically (Table 1).

**Table 1:** Demographics of the study population of 12,012 patients with a tibial plateau fracture

Patient characteristics	TKA	No TKA	Total
Number of patients	275	11,737	12,012
Age (mean, SD)	63.5 ± 11.4	56.4 ± 18.1	56.5 ± 18.1
Age groups			
< 55	62	5077	5139
55–64	76	2616	2692
65–74	92	2080	2172
75–84	37	1345	1382
≥ 85	8	619	627
Sex (%)			
Women	197 (71.6)	7343 (62.6)	7540 (62.8)
Type of injury			
“Low”	173 (63.0)	7660 (65.3)	7833 (73.3)
Mechanism of injury			
Transportation	63 (22.9)	2708 (23.0)	2771 (23.1)
Fall	168 (61.0)	7290 (62.1)	7458 (62.1)
Stress fracture	9 (3.3)	73 (0.6)	82 (0.7)
Other cause	35 (12.7)	1666 (14.2)	1701 (14.2)
AO/OTA classification (%)			
B1	78 (28.4)	3757 (33.7)	3957 (33.8)
B2	50 (18.1)	3291 (27.7)	3291 (28.0)
B3	63 (22.9)	1938 (16.6)	1938 (16.5)
C1	6 (2.2)	719 (6.2)	719 (6.1)
C2	15 (5.5)	428 (3.7)	428 (3.6)
C3	63 (23.0)	1404 (12.1)	1404 (12.0)

### Conversion to total knee arthroplasty

**Fig. 5:**
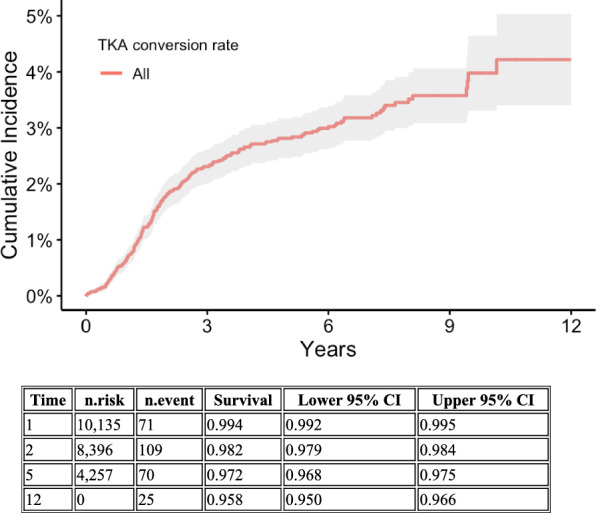
Kaplan–Meier graph showing total knee arthroplasty conversion in our population over time, with time on *x*-axis and cumulative percentage of conversion on *y*-axis. Including table that shows *n* at risk for different timepoints

In total, 275 of the 12,012 patients underwent a TKA within a follow-up period ranging from 0 to 12 years, with a mean follow-up of 4.2 years. Cumulative incidence of conversion at 5 years was 2.8%. Cumulative incidence of conversion at defined time points is presented in Table 3 and illustrated in Fig. 5.

### Fracture severity and conversion to TKA

**Fig. 6:**
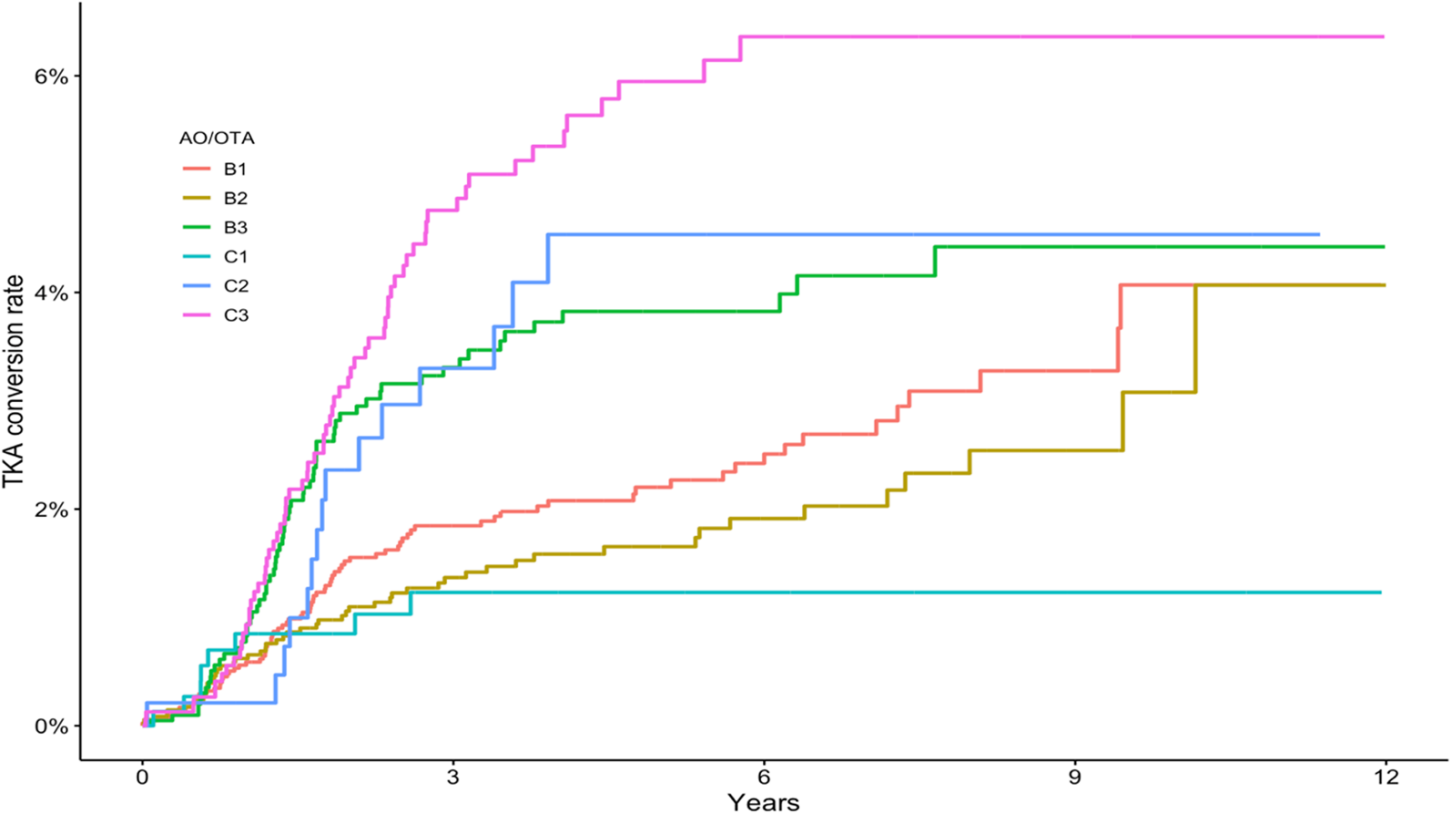
Kaplan–Meier survival analysis of conversion to total knee arthroplasty by fracture type. Time to conversion on the *x*-axis

We found a higher risk of TKA conversion in patients with comminuted fracture patterns AO/OTA 41B3, AO/OTA 41 C2, and AO/OTA 41 C3 compared with AO/OTA B1, with adjusted hazard ratios (aHRs) of 2.1 (95% CI 1.3–3.3), 2.3 (1.2–4.5) and 3.2 (95% CI 2.0–4.5), respectively, after adjusting for age, sex, and injury energy type. Conversion to TKA was performed faster in patients with B3, C2, and C3 fractures. Patients with B1 and B2 fractures displayed a slower gradual conversion over a 5-year period (Fig. 6) (Table 2).

**Table 2:** TKA conversion rate in 12,012 patients with tibial plateau fracture by initial fracture class according to AO/OTA, with primary nonoperative treatment or fracture fixation

Time (years)	B1 (95% CI)	B2 (95% CI)	B3 (95% CI)	C1 (95% CI)	C2 (95% CI)	C3 (95% CI)
1	0.6% (0.3–0.8)	0.6% (0.3–0.9)	0.9% (0.4–1.4)	0.8% (0.1–1.5)	0.2% (0.0–0.6)	1.0% (0.4–1.4)
2	1.5% (1.0–2.0)	1.1% (0.7–1.5)	2.9% (2.0–4.0)	0.8% (0.1–1.5)	2.4% (0.9–3.8)	3.1% (2.0–4.2)
5	2.2% (1.5–2.9)	1.7% (1.1–2.2)	3.8% (2.8–4.8)	1.2% (0.4–2.4)	4.5% (2.2–6.8)	6.0% (4.5–7.5)
12	4.1% (2.5–5.7)	4.1% (1.6–6.5)	4.5% (3.3–5.7)	1.2% (0.4–2.4)	4.5% (2.2–6.8)	7.2% (4.8–9.6)

Event probability in percent with 95% confidence intervals (CI)

### Patient and fracture characteristics associated with conversion to TKA

**Fig. 7:**
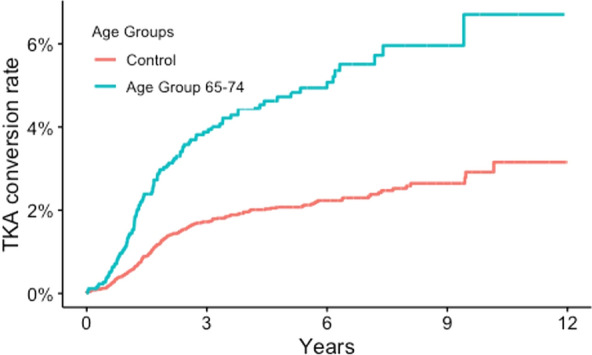
Kaplan–Meier survival analysis of age group associated with joint failure, with time to conversion to TKA on the *x*-axis. Comparing age group 65–74 with all other age groups

The conversion rate at 5 years varied by age group: 1.4% in patients younger than 55 years, 3.7% in patients aged 55–64 years, 5.1% in those aged 65–74 years, 3.3% in patients aged 75–84 years, and 1.9% in patients aged 85 years or older. Increasing age was also identified as a risk factor, with the 65–74 age group showing the highest risk of conversion (aHR = 3.7, 95% CI 2.6–5.1). Patients older than 75 years exhibited a progressively lower risk of conversion, with those aged 85 or older showing no significant difference compared with the control group (patients younger than 55 years). Sex was not a significant risk factor in our analysis (Table 3, Fig. 7).

**Table 3:** Risk of conversion to TKA, within 5 years of initial surgery and risk of conversion during the full follow-up. Hazard ratios (95% confidence intervals)

Group	5 years aHR (95% CI)	12 years aHR (95% CI)
AO/OTA 41B3	2.1 (1.3–3.3)	1.7 (1.1–2.6)
AO/OTA 41 C3	3.2 (2.0–5.0)	2.6 (1.7–3.9)
AO/OTA 41 C2	2.3 (1.2–4.5)	1.8 (1.0–3.4)
Age < 55 (reference)	–	–
Age 55–64	2.8 (1.9–4.0)	2.5 (1.7–3.5)
Age 65–74	3.7 (2.5–5.4)	3.6 (2.5–5.0)
Age 75–84	2.8 (1.7–4.5)	2.7 (1.7–4.2)
Age ≥ 85	1.8 (0.9–4.3)	1.7 (0.7–3.8)

High-energy trauma was not significantly associated with an increased risk of conversion to TKA with an aHR of 1.3 (95% CI 0.9–1.7).

In surgically treated patients, the cumulative incidence of conversion was 0.6% at 1 year, 2.5% at 2 years, 4.1% at 5 years, and 5.1% 12 years follow-up. In the nonsurgically treated group, the conversion rate was 1.7% at 5 years and 3.4% at full follow-up (Table 4). Fractures deemed serious enough to mandate surgical treatment were associated with an increased risk of conversion to TKA (aHR 1.8, 95% CI 1.3–2.5).

**Table 4:** Cumulative incidence of conversion rate to total knee arthroplasty

Time (years)	Operatively treated patients (event probability, 95% CI)	Nonoperatively treated patients (event probability, 95% CI)	All patients (event probability, 95% CI)
1	0.6% (0.4–0.8)	0.7% (0.5–0.9)	0.6% (0.5–0.8)
2	2.5% (2.0–2.9)	1.2% (0.9–2.0)	1.8% (1.5–2.1)
5	4.1% (3.5–4.7)	1.7% (1.3–2.0)	2.8% (2.5–3.2)
12	5.1% (4.0–6.2)	3.4% (2.2–4.6)	4.2% (3.4–5.0)

Event probability in percent with 95% confidence intervals (CI)

Surgeon experience did not influence conversion risk in our population.

### Sensitivity analysis

**Fig. 8:**
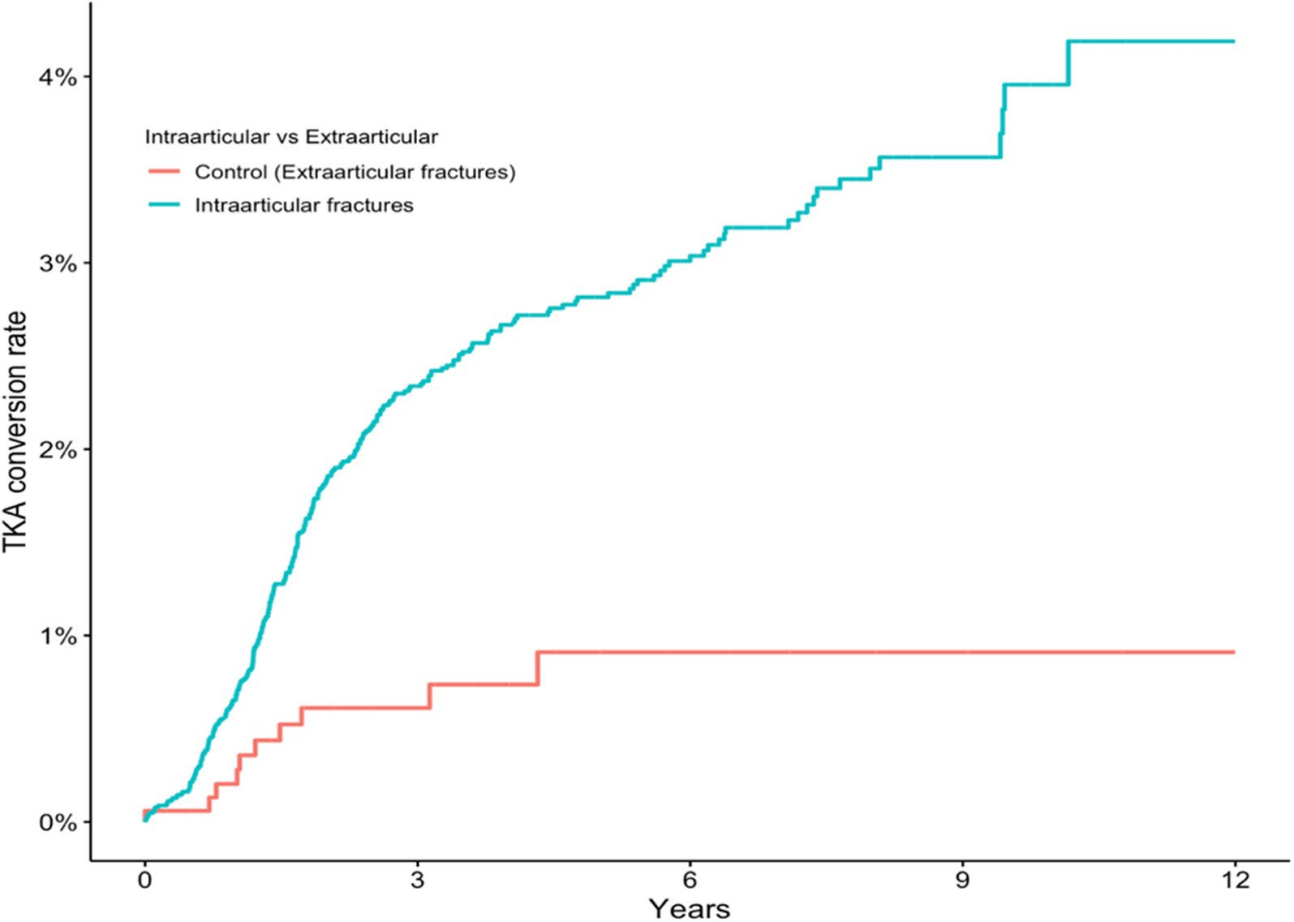
Kaplan–Meier graph showing risk of conversion to total knee arthroplasty for intraarticular fractures AO/OTA 41B-C in comparison with extraarticular fractures (AO-OTA 41 A, uninjured joint surface) to act as a control group

A total of 1608 patients had an extraarticular fracture of the proximal tibia (type A). The conversion rate of this “control group” was 0.2% a 1 year, 0.6% at 5 years, and 0.9% at 12 years follow-up. Intraarticular fracture patterns (type B and C., i.e., TPFs) were associated with an increased risk of conversion to TKA ([aHR = 3.3 (95% CI 1.7–6.2)] when compared with type A fractures (Fig. 8).

## Discussion

### Conversion to total knee arthroplasty

In this large register-based national observational study comprising 12,012 TPFs with primary surgical or nonsurgical treatment, we found a conversion rate to TKA of 2.8% at 5-year follow-up. To our knowledge this is the largest study to date investigating the risk of TKA conversion after a TPF, incorporating preoperative fracture classification as one of the most important confounders. With the aid of the SFR we managed to gather a substantial number of patients to include in the study. When registering a patient into the SFR, multiple variables are entered, such as mechanism of injury and fracture classification according to AO/OTA, making it a unique and valuable source of data for this type of study. Moreover, the linking to the SAR [13] resulted in the best possible control of the conversion to TKA, with high national completeness.

Our results of a national TKA conversion rate after TPF of 4.2% during full follow-up and 2.8% at 5-year follow-up is slightly lower than previously published material [1, 3, 4, 15, 22, 23]. This discrepancy can be partially attributed to the configuration of fracture characteristics. In our study, a significant majority of patients (71%) had simple articular fractures, i.e., classified as AO/OTA 41B1-2 and C1-2, with only 47% requiring surgical treatment. The fact that we have evaluated a national cohort of patients with both primary nonsurgical and surgical treatment, originating from different hospitals with varying treatment algorithms, may contribute to differences when compared with many publications that based their experiences on cohorts originating from specialized single centers.

The incidence of PTOA has been suggested to rise with increasing Schatzker classification [9], and the risk of conversion to TKA also escalates with greater comminution of TPFs [18]. Furthermore, more complex fractures are more likely to be treated surgically, which may explain the observed differences in outcomes.

Elsoe et al. studied a cohort from the Danish Patient Register comprising 7950 TPFs with no information on subsequent primary treatment and found a conversion rate to TKA of 5.7% with a mean follow-up of 13.9 years [5]. In another study using the Finnish hospital discharge register, a conversion rate to TKA of 4.4% was reported in 7701 patients after TPF, with a mean follow-up of 5.1 years [23]. Among the surgically treated group in that study, the conversion rate was 5.0%.

In a single-center study of 220 patients aged 60 years and older with TPFs, of which 40% were treated surgically, the total conversion rate to TKA was reported to be 8% [4].

A multicenter study from the Netherlands approached 862 patients with a surgically treated TPF and found a reported conversion rate of 14%, however, only 55% responded [1]. Wasserstein et al., using billing codes from the Ontario area in Canada, studied 8426 cases of surgically treated TPFs and found a conversion rate of 7.3% at 10-year follow-up [25].

Multiple studies have investigated the occurrence of radiographic PTOA after TPF, reporting rates that range widely from 17% to 73%. Even higher rates have been observed for bicondylar fractures [3, 6, 8, 9, 15, 20, 22]. However, we consider the TKA conversion rate to be a more meaningful metric than radiographic osteoarthritis, as it reflects not only structural changes, but also patient symptoms, functional limitations, and healthcare resource utilization.

While other studies have reported on large cohorts of patients with TPFs, they were not able to assess individual fracture characteristics or injury mechanisms.

Although studies that analyze individual medical records and radiographic data are preferred, they often lack the statistical power needed to draw valid conclusions regarding initial patient and fracture characteristics as factors associated with outcomes, due to the small number of TKA conversions, and loss to follow-up within the specified time span [1, 4, 18]

### Fracture severity and conversion to total knee arthroplasty

Our data clearly show an association between conversion rate to TKA and fracture type. More complex fractures according to the AO/OTA classification with a more severe articular injury, or comminuted joint surface, were associated with a higher likelihood of being converted to a TKA during the study period. The displacement and potential instability of the fracture are variables that may affect decision of treatment strategy, which is not captured in the fracture classification. Fractures with a primary surgical treatment were associated with a higher conversion rate compared with those treated nonsurgically. This may not be surprising, however, the fact that surgically treated fractures were not associated with an even higher conversion rate is somewhat surprising.

### Patient and fracture characteristics associated with conversion to TKA

In our model, age group was a significant risk factor for conversion to TKA up to 84 years. We observed a gradual increase in the risk of TKA conversion with advancing age, peaking in the 65–74 age group, where the highest conversion rate (5.1% at 5 years) was recorded. This suggests that presence of osteoporosis, degenerative joint changes, or delayed healing may contribute to the higher likelihood of joint failure in this age group. Unfortunately, our registry study design prevented us from assessing the degree of pre-fracture osteoarthritis on radiographs. Thus, our finding of the highest likelihood of conversion to TKA after a TPF among those aged 65–74 years must be considered in the context that this is also the most common age group undergoing TKA for degenerative disease. However, beyond the age of 75 years, the risk of conversion decreased, with patients aged 85 years or older showing no significant difference in risk compared with younger patients under 55 years. This could indicate that older patients might be less likely to undergo TKA due to a combination of factors such as lower physical demand, increased surgical risk, or different treatment approaches in elderly populations. Another possible explanation for the decrease in risk among the elderly population is that death acts as a competing variable; due to their age, death is more likely to occur before a potential conversion. Thus, the more accurate measurement of cumulative incidence was used, as it takes into account time under risk.

Sex and type of trauma (high/low energy) was not significantly associated with conversion to TKA.

### Strengths and limitations

**Fig. 9:**
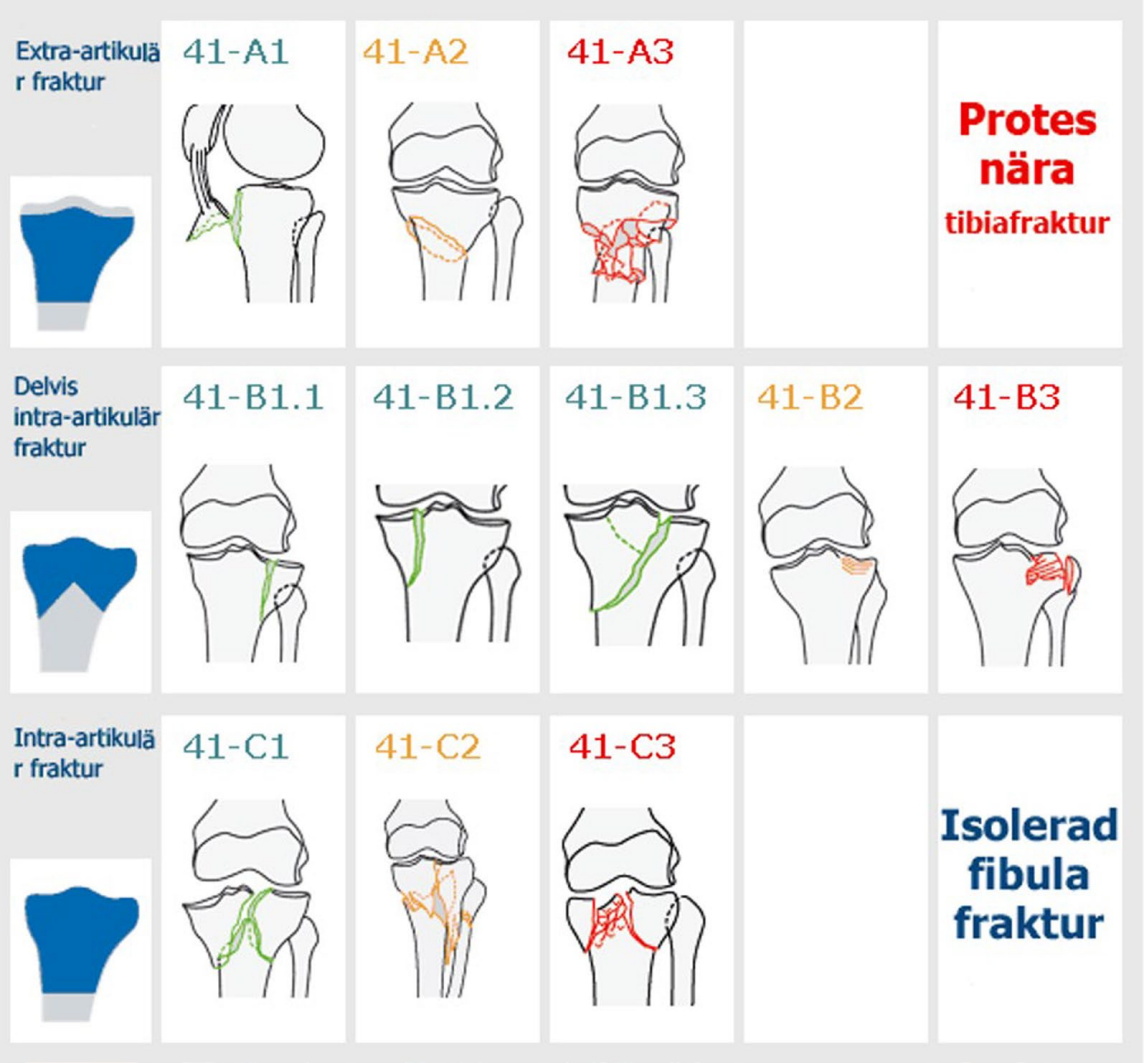
AO/OTA 2007 classification used in the Swedish Fracture Register. B- and C-type fractures included in this study as tibial plateau fractures

A limitation of our study is that fracture classification according to AO/OTA guidelines is dependent on the treating physician’s judgment. Although the SFR facilitates accurate data entry through an intuitive interface that incorporates AO/OTA classification images, ensuring proper registration, this process remains subjective (Fig. 9). Additionally, since the data are anonymized and preoperative radiographs are unavailable, verifying intraobserver agreement was not feasible. This restricts the ability to assess consistency in classification across different physicians. However, multiple validation studies in the SFR have demonstrated acceptable kappa values for intraobserver agreement, suggesting that the overall reliability of the data remains robust [11, 26]. Wennergren et al. [26] conducted a validation study on the classification of tibial fractures in the SFR and found a substantial agreement according to interpretations of kappa values set by Landis and Koch [12].

The decision to primarily treat fractures surgically or nonsurgically is influenced by multiple factors, including criteria not explicitly recorded in the Swedish Fracture Register and therefore could not be accounted for in the analysis. As a result, the division between surgical and nonsurgical treatment groups definitively introduces selection bias, which may affect the observed differences in TKA conversion rates. This limitation should be considered when interpreting the results.

Another limitation to this study is the hypothesis that a primary reason for TKA conversion after open reduction and internal fixation (ORIF) is the quality of bone, specifically the degree of osteoporosis. In patients with osteoporosis, a comminuted joint surface may be surgically treated successfully, and anatomical alignment of the joint surface can be restored perioperatively. However, poor bone quality may not withstand weight bearing, leading to loss of reduction over time. Thus, the lack of access to medical records and radiographic data limits our ability to determine whether manifest osteoporosis was present in these patients at the time of injury, or whether the fracture was successfully reduced and appropriately addressed with fixation.

The large number of subjects in our study, along with the comprehensive data on mechanisms of injury available through the SFR, helps to partially mitigate these limitations.

Another limitation to our study is the important question of whether the rate of conversion differs from the anticipated conversion in a healthy control representing the population in general, and to what degree. A number of patients initially identified but excluded due to their fracture being extraarticular were analyzed separately as a control. However, as these patients have sustained an injury to the knee and the rate of soft-tissue damage is unknown to us, they are rendered an unsuitable healthy control group.

Wasserstein et al. matched each member of their surgical cohort with four healthy individuals and found a natural TKA rate in the controls of 0.29%, 0.82%, and 1.8% after 2, 5, and 10 years, respectively [25].

The SFR and the SAR, with its high completeness and coverage, provide the largest cohort studied to date, offering more comprehensive follow-up compared with other registries or cohorts where endpoint (conversion to TKA) uncertainty may remain [1, 4, 25]. In 2023, the completeness for the SFR was 62.1% for tibia fractures, including ankle fractures, when compared with the National Patient Register, which on the contrary, over-exaggerates the true number of fractures [2]. This means that not all TPFs are included, but the ones that are have a fracture classification, and the conversion to TKA is well controlled by linkage to the SAR. The SAR is a well-established and reliable source for tracking conversions, as it includes all knee arthroplasties performed in Sweden, irrespective of the underlying diagnosis [13].

### Meaning of the study

Through this study, we aimed to gain insights into the preoperative factors that may influence outcomes and the risk of developing symptomatic PTOA, which would necessitate conversion to TKA. However, our results did not identify a specific group where the individual risk of joint failure was markedly elevated.

As this is a register-based study, we are limited by the available variables, for example, using the AO/OTA fracture classification registered by the treating orthopedic surgeon.

It may be necessary to reconsider how we classify TPFs if we aim to use these classifications as predictors for joint failure. In clinical practice, the gold standard involves performing a preoperative computed tomography (CT) scan to assess fracture patterns and to use that to construct a plan for approach and fracture fixation, should surgical treatment be chosen. Utilizing a 3D tool for mapping fractures could provide more options for classification. Cong-Feng Lou and colleagues have proposed a three-column classification system, now also included in the updated Schatzker classification [10], which primarily aids in preoperative planning [14]. However, none of the existing classifications widely in use, including Schatzker, AO/OTA, or the three-column system, account for the degree of comminution or depression of the articular surface. This limitation renders them somewhat blunt instruments when used as predictors of failure, as we now know that degree of comminution and level of joint surface depression are risk factors for joint failure[1, 4, 18].

## Conclusions

In this national register-based observational study, the conversion rate to TKA was 2.8% within 5 years of the primary injury and 5.1% at 12 years. Patients who underwent surgical treatment had a TKA conversion rate of 4.1% at 5 years, compared with 1.7% for those treated nonsurgically. Complex articular fractures (AO B3, C2, and C3) and patients aged 65–74 years were found to be at the highest risk of requiring TKA after a TPF.

## Data Availability

Data from this study are not freely available. However, data can be extracted from the national registries with an approved ethical application due to the sensitive nature of data from national quality registers.

## Abbreviations

aHR: Adjusted hazard ratio
AO/OTA: Arbeitsgemeinschaft für Osteosynthesefragen/Orthopaedic Trauma Association
KM: Kaplan–Meier survival analysis
ORIF: Open reduction internal fixation
PTOA: Post-traumatic osteoarthritis
SAR: Swedish Arthroplasty Register
SFR: Swedish Fracture Register
TKA: Total knee arthroplasty
TPF: Tibial plateau fracture
